# Clinical Characteristics and Predictors of Chronicity in Children With Immune Thrombocytopenia

**DOI:** 10.7759/cureus.105588

**Published:** 2026-03-21

**Authors:** Yara Alali, Ehab Hanafy, Mohammed Mustafa, Fadwa Abufara, Shimaa El-Shereif

**Affiliations:** 1 Pediatrics, King Salman Armed Forces Hospital, Tabuk, SAU; 2 Hematology and Oncology, Armed Forces Hospital Southern Region, Khamis Mushayt, SAU; 3 Oncology, King Salman Armed Forces Hospital, Tabuk, SAU

**Keywords:** chronic itp, chronicity prediction, early treatment response, itp, pediatric immune thrombocytopenia, platelet count, prognostic factors, saudi arabia

## Abstract

Introduction

Immune thrombocytopenia (ITP) in children is typically self-limited; however, a subset progresses to chronic disease. Identifying early predictors of chronic evolution remains clinically important to improve risk stratification and management strategies.

Methods

We conducted a retrospective cohort study of 47 children aged 1-14 years diagnosed with primary ITP at a tertiary care center between 2020 and 2025. Baseline demographic, clinical, laboratory, and treatment data were extracted from electronic records. Early treatment response was categorized as complete, partial, transient, or no response. Associations with chronic disease were evaluated using non-parametric testing and exact categorical analyses. Multivariable logistic regression was performed to identify independent predictors of chronicity.

Results

Chronic ITP developed in 12 of 47 patients (25.5%). The early response category was strongly associated with chronic evolution (p < 0.001). Among chronic patients, 91.7% exhibited a transient response pattern, and none achieved a sustained complete response. Baseline platelet count differed significantly between complete and non-complete responders (p = 0.002) and showed a near-significant association with chronicity in multivariable analysis (adjusted OR 1.057 per 1 ×10^3^/µL increase; 95% CI 0.997-1.120; p = 0.065). Age at diagnosis and autoimmune markers were not independently associated with chronic disease. The whole exome sequencing category demonstrated a significant association with chronicity (p < 0.001), though testing was clinically selected.

Conclusion

Early treatment response dynamics strongly discriminate long-term disease trajectory in pediatric ITP. Transient response is highly predictive of chronic evolution, whereas sustained complete response appears protective. Baseline platelet count may contribute to risk stratification. These findings support incorporating early response patterns into prognostic assessment models for pediatric ITP.

## Introduction

Immune thrombocytopenia (ITP) is the most common acquired autoimmune bleeding disorder in childhood and is defined by isolated thrombocytopenia in the absence of other identifiable causes of low platelet count [1]. The disorder is driven by immune-mediated platelet destruction together with impaired platelet production, resulting from autoantibody formation against platelet antigens and broader dysregulation of humoral and cellular immune pathways [2]. These mechanisms contribute not only to accelerated peripheral platelet clearance but also to impaired megakaryocyte function, thereby shaping the clinical spectrum of disease [3].

The reported incidence of pediatric ITP ranges from approximately two to five cases per 100,000 children annually, making it a frequent hematologic diagnosis in pediatric practice [4]. Children typically present with petechiae, purpura, ecchymosis, or mucosal bleeding, while severe hemorrhagic manifestations remain uncommon. In most cases, the disease follows a favorable and self-limited course, with spontaneous remission occurring within the first months after diagnosis [5].

In pediatric patients, ITP often follows viral infection or other forms of immune stimulation, supporting the concept that transient immune dysregulation plays an important role in disease onset [5,6]. Nevertheless, the clinical course is heterogeneous. While many children recover completely, a clinically meaningful subset develops persistent or chronic thrombocytopenia. According to international consensus criteria, chronic ITP is defined by thrombocytopenia lasting more than 12 months after diagnosis [1,7].

Previous studies have shown that nearly one-fifth to one-third of children with ITP progress to chronic disease, emphasizing the need for reliable early predictors of long-term outcome [7,8]. This distinction is clinically relevant because chronic disease may require prolonged monitoring, repeated interventions, and sustained healthcare utilization, with potential effects on quality of life for both patients and families [9].

A range of demographic, clinical, and laboratory variables has therefore been examined as possible predictors of chronicity, including age at diagnosis, sex, baseline platelet count, bleeding severity, treatment exposure, and immune-related markers [10,11,12]. Among these, early response to treatment has attracted particular interest as a potentially practical prognostic indicator. Several studies suggest that children who achieve rapid and sustained platelet recovery are more likely to enter long-term remission, whereas those with transient or incomplete recovery may be at increased risk for persistent disease [10,12,13].

At the same time, advances in the understanding of ITP biology have reinforced the multifactorial basis of chronic disease [2]. Autoantibody-mediated platelet destruction, impaired megakaryocyte maturation, cytotoxic T-cell activity, and abnormalities in immune regulation have all been implicated in disease persistence [3,14,15]. These mechanistic insights highlight why clinical trajectories may differ substantially between patients who initially appear similar at presentation.

Despite these advances, the prediction of chronic evolution in pediatric ITP remains imperfect, particularly in routine clinical settings where treatment approaches, investigation strategies, and follow-up patterns vary. Further studies examining real-world pediatric cohorts are therefore valuable to clarify which early characteristics are most strongly associated with later chronicity.

Accordingly, the aim of the present study was to evaluate the clinical characteristics and predictors of chronic ITP in children treated at a tertiary care center, with particular emphasis on early response patterns and their relationship to subsequent chronic disease.

## Materials and methods

Study design and setting

This retrospective observational cohort study was conducted at King Salman Armed Forces Hospital, Tabuk, Saudi Arabia. Pediatric patients diagnosed with primary ITP between January 2020 and December 2025 were identified through electronic medical records. The study was approved by the Institutional Research Ethics Committee, King Salman Armed Forces Hospital (approval no.: KSAFH-RET-2025-633), and all data were handled in accordance with ethical and confidentiality standards.

Study population

Children aged 1-14 years diagnosed with primary ITP according to established international diagnostic criteria were eligible for inclusion. ITP was classified based on disease duration as newly diagnosed (<3 months), persistent (3-12 months), or chronic (>12 months) [11]. Patients with secondary causes of thrombocytopenia, including hematologic malignancies, bone marrow failure syndromes, or systemic autoimmune disorders, were excluded. Patients with incomplete clinical or follow-up data were also excluded. A total of 47 patients met eligibility criteria and were included in the final analysis.

Data collection

Clinical and laboratory data were extracted systematically from electronic records. Baseline variables included age at diagnosis, sex, presenting symptoms, severity at presentation, history of preceding infection, vaccination history, platelet count at diagnosis, and whether bone marrow aspiration (BMA) was performed. The initial management strategy was categorized as observation, intravenous immunoglobulin (IVIG), or corticosteroids.

Early treatment response was classified into four categories: complete response, partial response, transient response, and no response, based on platelet recovery dynamics during follow-up. Complete response was defined as sustained normalization of platelet count without relapse during the defined observation period. The current platelet count at follow-up was recorded for outcome comparison.

Autoimmune markers, including antinuclear antibody (ANA), anti-double-stranded DNA (anti-dsDNA), complement component 3 (C3), and complement component 4 (C4), were recorded when available. Whole exome sequencing (WES) results were categorized as not performed, normal, or pathogenic variant detected.

Outcome measures

The primary outcome was the development of chronic ITP. Secondary outcomes included early treatment response patterns and current platelet count at follow-up.

Statistical analysis

Statistical analysis was performed using IBM SPSS Statistics for Windows, Version 27 (Released 2019; IBM Corp., Armonk, New York, United States). Continuous variables were assessed for distribution and summarized as median and interquartile range (IQR) due to non-normal distribution. Categorical variables were expressed as frequencies and percentages.

Comparisons between two independent groups for continuous variables were performed using the Mann-Whitney U test. Comparisons across more than two groups were conducted using the Kruskal-Wallis test. Associations between categorical variables were assessed using Pearson chi-square testing. When expected cell counts were small, exact methods were applied, including Fisher’s exact test or the Fisher-Freeman-Halton extension for larger contingency tables. A two-sided p-value < 0.05 was considered statistically significant.

Multivariable analysis

To identify independent predictors of chronic disease, multivariable logistic regression analysis was performed. Variables entered into the model were selected based on clinical relevance and univariate associations, including platelet count at diagnosis, age at diagnosis, and early complete response status. Adjusted odds ratios (ORs) with 95% confidence intervals (CIs) were calculated.

Model performance was evaluated using the Omnibus test of model coefficients, Nagelkerke R^2^ to estimate explained variance, and the Hosmer-Lemeshow goodness-of-fit test to assess calibration. Overall classification accuracy was also reported. In the presence of complete separation, specifically, the absence of chronic disease among patients achieving complete response, regression estimates were not computable and were reported as non-estimable.

## Results

A total of 47 pediatric patients were included in the final analysis, with complete baseline, treatment response, and follow-up data available for all cases. At presentation, 29 (61.7%) had newly diagnosed ITP, six (12.8%) had persistent ITP, and 12 (25.5%) met criteria for chronic ITP. The median age at diagnosis was five years (IQR 5-9), and the median platelet count at diagnosis was 14 ×10^3^/µL (IQR 4-14). Most patients were symptomatic at presentation, occurring in 43 (91.5%), most commonly with petechiae, ecchymosis, and/or epistaxis. A preceding history of infection was documented in 42 (89.4%), while severe presentation at diagnosis was uncommon, occurring in one (2.1%). No patient had a recent history of vaccination; therefore, vaccination could not be evaluated as an associated factor in this cohort. BMA was performed in 12 (25.5%) and was not performed in 35 (74.5%); among chronic patients who underwent BMA, results were reported as normal. Baseline demographic and clinical characteristics are summarized in Table 1.

**Table 1:** Baseline demographic and clinical characteristics of the cohort (N = 47) Values are n (%) unless otherwise stated. Age and platelet count are presented as median (IQR). BMA: bone marrow aspiration; WES: whole exome sequencing; ITP: immune thrombocytopenia; IVIG: intravenous immunoglobulin

Variable		Value
Age at diagnosis, years		5 (5-9)
Male sex		30 (63.8)
Platelet count at diagnosis (×10^3^/µL)		14 (4-14)
Symptomatic patients		43 (91.5)
Severe presentation		1 (2.1)
History of infection		42 (89.4)
History of recent vaccination		0 (0)
BMA performed		12 (25.5)
ITP classification	Newly diagnosed	29 (61.7)
	Persistent ITP	6 (12.8)
	Chronic ITP	12 (25.5)
Initial treatment	Observation	7 (14.9)
	IVIG	38 (80.9)
	Steroids	2 (4.3)
WES finding	Not done	31 (66)
	Normal	5 (10.6)
	Pathogenic variant detected	11 (23.4)

A strong association was observed between the early treatment response category and subsequent chronic disease evolution (χ^2^ = 26.64, df = 3, p < 0.001) (Table 2). Among patients who developed chronic disease, 11 (91.7%) demonstrated a transient response pattern, and none achieved a sustained complete response. In contrast, among non-chronic patients, 15 (42.9%) achieved a complete response, whereas four (11.4%) exhibited a transient response. These findings indicate that early response dynamics strongly discriminate long-term disease trajectory.

**Table 2:** Association between early response category and chronic ITP Pearson chi-square test with exact significance (Fisher-Freeman-Halton) applied due to small expected cell counts. ITP: immune thrombocytopenia

Response category	Non-chronic (n = 35)	Chronic (n = 12)	Test statistic	p-value
Complete	15 (42.9%)	0 (0.0%)	-	-
Partial	15 (42.9%)	1 (8.3%)	-	-
Transient	4 (11.4%)	11 (91.7%)	-	-
None	1 (2.9%)	0 (0.0%)	-	-
Overall association	-	-	χ^2^ = 26.64	<0.001

Baseline platelet count at diagnosis differed significantly between patients who achieved complete response and those who did not (Mann-Whitney U = 105.0, Z = -3.087, p = 0.002) (Table 3). The median platelet count at diagnosis was higher among complete responders (41 ×10^3^/µL, IQR 3-80) than among non-complete responders (11 ×10^3^/µL, IQR 1-21). In contrast, age at diagnosis did not differ significantly by complete response status (p = 0.304), and neither history of infection (p = 0.309), severe presentation (p = 1.000), nor BMI (p = 0.380) showed a significant association with complete response (Table 3).

**Table 3:** Factors associated with complete response (complete vs. non-complete) Continuous variables were compared using the Mann-Whitney U test and are presented as median (IQR). Categorical variables were compared using Fisher’s exact test where appropriate. The corresponding test statistic is presented as U for the Mann-Whitney U test; for Fisher’s exact test, the test statistic is reported as NA.

Variable	No complete (n = 32)	Complete (n = 15)	Test used	Test statistic	p-value
Age at diagnosis, years, n (%)	7 (8)	8 (8)	Mann-Whitney U	U = 213	0.545
Platelet count at diagnosis (×10^3^/µL), n (%)	11 (20)	41 (77)	Mann-Whitney U	U = 105	0.002
Chronic disease, n (%)	12 (37.5)	0 (0)	Fisher’s exact test	NA	<0.001
History of infection, n (%)	30 (93.8)	12 (80.0)	Fisher’s exact test	NA	0.309
Severe presentation, n (%)	1 (3.1)	0 (0)	Fisher’s exact test	NA	1.000

The initial treatment strategy was significantly associated with achieving a complete response (χ^2^ = 18.42, df = 2, p < 0.001; Fisher-Freeman-Halton exact test, p < 0.001) (Table 4). Among patients managed initially with observation, seven (100.0%) achieved complete response, compared with seven (18.4%) treated with IVIG and one (50.0%) treated with steroids. Given the non-randomized treatment allocation and small subgroup sizes, these findings should be interpreted cautiously as associations rather than causal effects.

**Table 4:** Initial treatment modality and complete response Data are presented as n (%). P-value derived from the Fisher-Freeman-Halton exact test due to small expected cell counts. IVIG: intravenous immunoglobulin

Initial treatment	No complete response	Complete response	Test statistic	p-value
Observation	0 (0.0)	7 (100.0)	-	-
IVIG	31 (81.6)	7 (18.4)	-	-
Steroids	1 (50.0)	1 (50.0)	-	-
Overall association	-	-	χ^2^ = 18.42	<0.001

Current platelet counts at follow-up differed significantly across response categories (Kruskal-Wallis H = 22.18, df = 3, p < 0.001) (Table 5). Patients with transient response demonstrated substantially lower median current platelet counts (79 ×10^9^/L, IQR 49-125.5) than complete responders (287 ×10^9^/L, IQR 144-365) and partial responders (305.5 ×10^9^/L, IQR 252.5-353). The no-response category included one (2.1%) patient and was not suitable for distributional inference.

**Table 5:** Current platelets count by response category Values are presented as median (IQR). P-value derived from the Kruskal-Wallis test. The corresponding test statistic is reported as H. The no-response category was not included in distributional inference because the sample size was insufficient (n = 1).

Response category	N	Current platelet count (×10^9^/L)	Test statistic	p-value
Complete	15	287 (144-365)	-	-
Partial	16	305.5 (252.5-353)	-	-
Transient	15	79 (49-125.5)	-	-
No response	1	Not calculated	-	-
Overall comparison	-	-	H = 22.18	<0.001

Autoimmune screening did not demonstrate a significant association with chronicity, including ANA status (χ^2^ = 2.315, df = 2, p = 0.314), anti-dsDNA (p = 0.703), C3 (p = 0.583), or C4 (p = 0.740) (Table 6).

**Table 6:** Autoimmune markers and complement levels according to chronic disease status Data are presented as n (%). Pearson chi-square test was used where applicable; Fisher’s exact test was applied when expected cell counts were <5. The corresponding test statistic is reported as χ^2^, where available; for Fisher’s exact test, the test statistic is reported as NA. ANA: antinuclear antibody; anti-dsDNA: anti-double-stranded DNA antibody; C3: complement component 3; C4: complement component 4

Variable	Category	Non-chronic	Chronic	Test statistic	p-value
ANA	Not done	6 (85.7)	1 (14.3)	NA	0.314
ANA	Normal	25 (69.4)	11 (30.6)	NA	0.314
ANA	Abnormal	4 (100.0)	0 (0.0)	NA	0.314
ANA overall association	NA	NA	NA	χ^2^ = 2.315	0.314
Anti-dsDNA	Not done	9 (81.8)	2 (18.2)	NA	0.703
Anti-dsDNA	Normal	26 (72.2)	10 (27.8)	NA	0.703
Anti-dsDNA overall association	NA	NA	NA	NA	0.703
C3	Not done	19 (79.2)	5 (20.8)	NA	0.583
C3	Normal	15 (68.2)	7 (31.8)	NA	0.583
C3	Abnormal	1 (100.0)	0 (0.0)	NA	0.583
C3 overall association	NA	NA	NA	NA	0.583
C4	Not done	18 (78.3)	5 (21.7)	NA	0.740
C4	Normal	17 (70.8)	7 (29.2)	NA	0.740
C4 overall association	NA	NA	NA	NA	0.740

WES status was significantly associated with chronicity (χ^2^ = 14.20, df = 2, p < 0.001) (Table 7). Chronic ITP was observed in three (9.7%) patients in whom WES was not performed, four (80.0%) of those with a normal WES result, and five (45.5%) of those with a pathogenic variant detected (Table 7). However, WES testing was clinically selected and therefore susceptible to indication bias.

**Table 7:** Whole exome sequencing (WES) category and chronicity Data are presented as n (%). The Pearson chi-square test was used; the Fisher-Freeman-Halton exact test was applied where appropriate.

WES result	Non-chronic	Chronic	Test statistic	p-value
Not done	28 (90.3)	3 (9.7)	-	-
Normal	1 (20.0)	4 (80.0)	-	-
Pathogenic variant	6 (54.5)	5 (45.5)	-	-
Overall association	-	-	χ^2^ = 14.20	<0.001

To evaluate independent predictors of chronic disease, a multivariable logistic regression model was constructed, including platelet count at diagnosis, complete response status, and age at diagnosis (Table 8).

**Table 8:** Multivariable logistic regression analysis for predictors of chronic disease (n = 47) Model statistics: Omnibus χ^2^ = 16.77 (df = 3), p < 0.001; Nagelkerke R^2^ = 0.442; Hosmer-Lemeshow χ^2^ = 3.418 (df = 7), p = 0.844; overall classification accuracy = 78.7%. * Complete response was not estimable due to complete separation in the dataset (no patient who developed chronic disease achieved a complete response). ORs are expressed per 1 × 10^3^/µL increase in platelet count. B: regression coefficient; SE: standard error; OR: odds ratio; CI: confidence interval

Variable	B	SE	Wald	Adjusted OR	95% CI	p-value
Platelet count at diagnosis (per 1 ×10^3^/µL)	0.055	0.030	3.39	1.057	0.997-1.120	0.065
Age at diagnosis (years)	0.091	0.105	0.75	1.095	0.891-1.347	0.388
Complete response	Not estimable*	-	-	-	-	-

The overall model was statistically significant (Omnibus test χ^2^ = 16.77, df = 3, p < 0.001), explaining 44.2% of variance in chronic disease status (Nagelkerke R^2^ = 0.442), with acceptable calibration (Hosmer-Lemeshow χ^2^ = 3.418, df = 7, p = 0.844) and an overall classification accuracy of 78.7%. Platelet count at diagnosis showed a near-significant trend toward association with chronicity (adjusted OR 1.057 per 1 ×10^3^/µL increase; 95% CI 0.997-1.120; p = 0.065), while age at diagnosis was not independently associated (adjusted OR 1.095; 95% CI 0.891-1.347; p = 0.388).

Complete response demonstrated statistical separation (no chronic patient achieved complete response), resulting in unstable/non-estimable effect estimates, further underscoring the dominant prognostic value of complete response in preventing chronicity (Table 8).

## Discussion

In this retrospective cohort of children with primary ITP, 25.5% of patients developed chronic disease. This proportion is in keeping with prior pediatric reports showing that approximately 20-30% of children with ITP progress to chronic ITP rather than undergoing complete spontaneous or treatment-associated resolution [4,8]. Our findings therefore reinforce the well-recognized heterogeneity of pediatric ITP and the clinical importance of identifying early prognostic indicators.

The most prominent finding in the present study was the strong association between the early response category and long-term disease trajectory. Transient response predominated among children who later developed chronic ITP, whereas no child with a sustained complete response progressed to chronic disease. This pattern is highly relevant clinically because it suggests that early platelet recovery dynamics may provide more meaningful prognostic information than baseline presentation alone. These observations are broadly consistent with previous literature indicating that the quality and durability of early response reflect underlying disease biology and may help distinguish children likely to remit from those at risk for persistence [10,13].

From a biological perspective, this association is plausible. Chronic ITP is increasingly understood as a disorder sustained by multiple interacting immune mechanisms, including antibody-mediated platelet destruction, impaired platelet production, T-cell dysregulation, and defective immune tolerance [3,14]. A transient rise in platelet count after initial therapy may therefore represent temporary suppression of immune-mediated platelet clearance without durable restoration of immune homeostasis. By contrast, sustained complete response may reflect a more favorable immunologic course, even when achieved early in the disease process [15].

Our results also showed that baseline platelet count at diagnosis differed significantly between patients with and without complete response, with complete responders presenting with higher platelet counts. In the multivariable model for chronic disease, platelet count showed a near-significant association, although it did not meet the conventional threshold for independent statistical significance. Taken together, these findings suggest that baseline platelet count may have prognostic relevance, but its role appears more nuanced than that of early response pattern. In this cohort, platelet count should therefore be interpreted as a potentially contributory marker rather than a standalone predictor of chronicity. This cautious interpretation is important, particularly because prior studies have reported variable associations between platelet count at presentation and long-term outcome [5,11].

Age at diagnosis was not independently associated with chronic disease in our cohort. This contrasts with some reports suggesting that older age may be linked to persistence, but is in line with other studies showing inconsistent or limited predictive value for age when examined alongside other clinical factors [5,9]. The lack of significance in our study may reflect the modest sample size, cohort-specific characteristics, or the stronger discriminative value of early response behavior compared with demographic variables.

Autoimmune markers, including ANA, anti-dsDNA, C3, and C4, were not significantly associated with chronicity in this analysis. This finding suggests that, within an otherwise typical pediatric ITP population, routine autoimmune serology may have limited utility for predicting chronic course. Although immune dysregulation is central to ITP pathophysiology [2], conventional serologic markers may not adequately capture the specific immunobiologic processes that determine disease persistence in most children.

In our cohort, BMI was not significantly associated with achievement of complete response (p = 0.380), suggesting that body habitus did not demonstrate a measurable influence on early treatment response in this dataset. This finding differs from the report by Hanafy and Pakra, who described obese children with ITP who appeared refractory to multiple therapeutic approaches and proposed a possible association between obesity and treatment resistance [16]. However, that report was based on a small case series and was intended to generate a hypothesis rather than establish a causal relationship. Accordingly, our findings do not support a clear adverse effect of BMI on early response, although the question remains clinically relevant and warrants evaluation in larger prospective cohorts [16].

An additional finding in our study was the statistically significant association between the WES category and chronicity. This observation, however, should be interpreted with considerable caution. In pediatric thrombocytopenia, exome sequencing is typically pursued in selected patients with atypical features, diagnostic uncertainty, suspected inherited thrombocytopenia, or an unusually persistent course, rather than as a routine investigation for all cases [17]. In our cohort, WES was likewise not performed systematically, making the observed association highly vulnerable to indication bias. It is therefore not possible to conclude that the WES category independently predicts chronic ITP; more plausibly, the result reflects preferential testing in children with more complex or atypical presentations.

We also observed a significant association between initial treatment modality and achievement of complete response, with all initially observed patients achieving complete response. This finding should not be interpreted as evidence that observation is superior to IVIG or corticosteroids as a therapeutic strategy. In childhood ITP, initial management is guided primarily by bleeding severity, clinical stability, and physician judgment, and current guidelines support observation for children with no or only minor bleeding [11]. Consequently, patients selected for observation often represent a clinically more favorable subgroup at baseline, whereas those treated with IVIG or steroids may have had more symptomatic or higher-risk presentations. Our result is therefore best understood as a non-randomized association shaped by treatment selection rather than a causal treatment effect [11,18].

Importantly, the multivariable model supported the overall prognostic structure suggested by the univariate analyses. The model demonstrated acceptable fit and classification performance, while the complete response produced statistical separation because no patient with a complete response developed chronic disease. In logistic regression, complete separation is a recognized problem in small datasets when one predictor perfectly predicts the outcome, leading to unstable or non-estimable coefficients despite a potentially strong underlying association. Thus, the non-estimable adjusted effect for complete response in our model should not be viewed as weakening its prognostic significance; rather, it statistically reinforces how strongly sustained complete response was linked to a favorable long-term outcome in this cohort [19].

Overall, the present study supports the early response pattern as the most clinically informative marker of future chronicity in pediatric ITP. In practical terms, children with transient early response may warrant closer follow-up and more cautious counseling regarding the risk of prolonged disease, whereas sustained complete response appears reassuring. These findings may help refine risk stratification in everyday practice and support a more individualized approach to monitoring after diagnosis.

Limitations

Our results should be interpreted in light of the study limitations. The retrospective design introduces the possibility of information bias, and the relatively small sample size limits power for detecting modest associations. The single-center setting may also reduce generalizability. In addition, several evaluated variables, particularly WES and autoimmune markers, were not uniformly assessed across all patients. Nevertheless, despite these constraints, the internal consistency of the findings, especially the clear signal associated with the early response pattern, supports the clinical relevance of the observed associations.

## Conclusions

Early response pattern appears to be the most clinically relevant predictor of chronicity in pediatric ITP in this cohort. Children with a transient early response were more likely to progress to chronic disease, whereas a sustained complete response was associated with a favorable long-term outcome.

These findings support the value of early treatment response as a practical tool for risk stratification and follow-up planning in children with ITP. In contrast, age, autoimmune markers, and BMI were not significant predictors in this study. Further large prospective multicenter studies are needed to validate these findings and refine the prediction of chronic disease in pediatric ITP.
